# The Impact of COVID-19 on Otolaryngology Community Practice in Massachusetts

**DOI:** 10.1177/0194599820983732

**Published:** 2021-09

**Authors:** Timothy Fan, Alan D. Workman, Lauren E. Miller, Mallory Mason Sakats, Karthik Rajasekaran, Jason A. Brant, Arjun K. Parasher, David Huckins, Avner Aliphas, Robin Glicksman, Antoine Eskander, Jordan T. Glicksman

**Affiliations:** 1 Texas A&M College of Medicine, Bryan, Texas, USA; 2 Department of Otolaryngology, Massachusetts Eye and Ear Infirmary, Boston, Massachusetts, USA; 3 Harvard Medical School, Boston, Massachusetts, USA; 4 ENT Billing Associates, Worcester, Massachusetts, USA; 5 Department of Otorhinolaryngology, University of Pennsylvania, Philadelphia, Pennsylvania, USA; 6 Leonard Davis Institute of Health Economics, University of Pennsylvania, Philidelphia, Pennsylvania, USA; 7 Department of Otolaryngology–Head and Neck Surgery, University of South Florida, Tampa, Florida, USA; 8 Newton-Wellesley Hospital, Newton, Massachusetts, USA; 9 Department of Otolaryngology–Head and Neck Surgery, Boston University Medical Center, Boston, Massachusetts, USA; 10 Faculty of Medicine, University of Toronto, Ontario, Canada; 11 Department of Otolaryngology–Head and Neck Surgery Sunnybrook Health Sciences Centre and Institute for Health Policy Management and Evaluation, Ontario, Canada

**Keywords:** COVID-19, coronavirus, health policy, statistics, clinical practice guidelines

## Abstract

**Objectives:**

Coronavirus disease 2019 (COVID-19) significantly affected many health care specialties, including otolaryngology. In response to governmental policy changes, many hospitals and private practices in Massachusetts canceled or postponed nonurgent office visits and elective surgeries. The objective of this study was to quantify the impact of COVID-19 on the provision and practice trends of otolaryngology services for 10 private practices in Massachusetts.

**Study Design:**

Retrospective review.

**Setting:**

Multipractice study for community practices in Massachusetts.

**Methods:**

Electronic billing records from 10 private otolaryngology practices in Massachusetts were obtained for the first 4 months of 2019 and 2020. Questionnaires from these otolaryngology practices were collected to assess financial and staffing impact of COVID-19.

**Results:**

The local onset of the COVID-19 pandemic had a significant decrease of 63% of visits in comparison to equivalent weeks in 2019. Virtual visits overtook in-person visits over time. A greater decline in operating room (OR) procedures than for office procedures was recorded. Ninety percent of practices reduced working hours, and 80% furloughed personnel. Seventy percent of practices applied for the Paycheck Protection Program (PPP).

**Conclusion:**

COVID-19 has had a multifaceted impact on private otolaryngology practices in Massachusetts. A significant decline in provision of otolaryngology services aligned with the Massachusetts government’s public health policy changes. The combination of limited personnel and personal protective equipment, as well as suspension of nonessential office visits and surgeries, led to decrease in total office visits and even higher decrease in OR procedures.

A severe acute respiratory syndrome coronavirus 2 (SARS-CoV-2) strain coronavirus, commonly known as coronavirus disease 2019 (COVID-19), emerged in Wuhan, China, in December 2019^1,2^ with rapid spread and significant morbidity and mortality. As a result, the World Health Organization (WHO) declared COVID-19 a pandemic, the first time since the H1N1 influenza A virus in 2009.^3^ Otolaryngology in particular is perceived as a high-risk field for exposure, given high COVID-19 viral titers in the nasopharynx and other nearby mucosal surfaces. This theoretical exposure risk was compounded by shortages of personal protective equipment (PPE) in the early phases of the pandemic, rendering many otolaryngologists potentially susceptible to COVID-19.^4^

The explosive growth of new COVID-19 cases overwhelmed health care systems worldwide, accompanied by a disruption in the provision of elective and semielective health care services across many medical specialties. In particular, the state of Massachusetts was profoundly affected by the COVID-19 pandemic, with case totals in the tens of thousands in the first 2 months alone,^5,6^ along with concurrent shortages of ventilators, staff, and PPE.^7^ Following national and local government recommendations, nonurgent clinical visits and elective surgical procedures were postponed or canceled across many institutional hospitals and private practices across Massachusetts. The impact of COVID-19 across medical and surgical specialties remains under investigation. Not only are data pertaining to changes in practice patterns due to COVID-19 lacking for otolaryngology, but the few available reports have all been generated by academic institutions. However, private practices comprise the majority of otolaryngology care delivery within the United States. In the present study, we use aggregated data from 10 otolaryngology private practices in Massachusetts to quantify the impact of COVID-19 on the provision of otolaryngology services and identify changes in practice patterns for private community practices.

## Materials and Methods

This is a retrospective review of private practice physician services provided at 10 private practices in Massachusetts. Institutional review board (IRB) approval was obtained for this study from the Partners Health Care IRB (IRB #2020P001248).

### Study Population

All Massachusetts-based private practice physicians using the services of ENT Billing Associates (an independent national medical billing services organization) between January 1, 2019, and April 30, 2020, were eligible for inclusion in this study. Physicians were excluded if they had not remained at the same practice throughout the entire study period.

### Data Collection

The electronic billing records of ENT Billing Associates were reviewed and compiled by week. Service and encounter data were determined based on billed Evaluation and Management (E/M) codes and *Current Procedural Terminology* (*CPT*) codes. Data were collected based on fiscal weeks beginning with the first Monday to Sunday period of each year (2019 and 2020) and extending for 16 weeks in total for that year. Encounters were determined based on any visits that generate an E/M *CPT* code or chart flag for a visit not designated to generate a bill (eg, a visit within a global period). Procedures were identified based on the most common procedures performed in these general otolaryngology practices and grouped by *CPT* code type, including endoscopy (31231, 31237, 31238, 31575, 31579), cerumen removal (69210, G0268), septoplasty (30520), tonsillectomy and/or adenoidectomy (42820, 42821, 42825, 42826, 42830, 42831), epistaxis management (30901, 30903, 30905, 30906), myringotomy with or without tubes (69420, 69421, 69433, 69436), and endoscopic sinus surgery (31254-31288). Patient visits were considered new if a new patient visit (99201-99205) or a consult (referral) code (99241-99245) was billed. All other E/M codes were considered return patient visits (99211-99215). A visit was considered “virtual” based on the Healthcare Common Procedure Coding System (HCPCS) modifiers (95, GT, GQ) and place of service (POS) modifiers and/or POS code, as well as telehealth audio codes (99441-99443).

In addition, a questionnaire was distributed to each practice. This was completed by either the practice manager or a representative physician from the practices. The questionnaire detailed the financial and staffing impact of COVID-19.

### Statistical Analysis

Comparisons were made between the early fiscal weeks (weeks 1-10) and late fiscal weeks (weeks 11-16) of the first third of 2019 and 2020. Specifically, *t* test comparisons were made between early and late 2019, early and late 2020, early 2019 and 2020, and late 2019 and 2020. This was performed using Stata software (StataCorp). GraphPad Prism version 8.0.2 (GraphPad Software) was used for visualization of data.

## Results

Demographic information was captured from 20 general otolaryngology physicians across 10 private practices in Massachusetts and is shown in **Table 1**. There was a mean (SD) of 2.1 (1.6) physicians per clinic, as well as a mean (SD) physician age of 51.4 years (7.8). Eight of 10 practices furloughed personnel, while 2 clinics experienced permanent layoffs. Cutbacks in working hours took place in 90% of practices. Among those that cut back hours, there was a mean (SD) reduction by 75.5% (12.1%). Seven clinics applied for the federal Paycheck Protection Program (PPP), all of whom reported reduced necessity for layoffs due to PPP utilization. Between weeks 11 and 16 of 2020, only 20% private practices provided audiology in person, and no practices were able to do so remotely. No practices provided any nonemergent audiology during this period. During these weeks, only 60% of practices continued performing endoscopy, with all of them implementing stricter selective criteria.

**Table 1.** Demographics of 10 Private Otolaryngology Practices in the Boston Area.

Characteristic	Value
Clinic characteristics, mean (SD)	
Number of physicians	2.1 (1.6)
Age of physicians, y	51.4 (7.8)
Distance from Boston, miles	32.3 (29.4)
Number of beds at associated hospital	253.3 (210.4)
Physician extenders, No. (%)	
Practices with a nurse practitioner	2 (20)
Practices with a physician assistant	4 (40)

**Figure 1.**
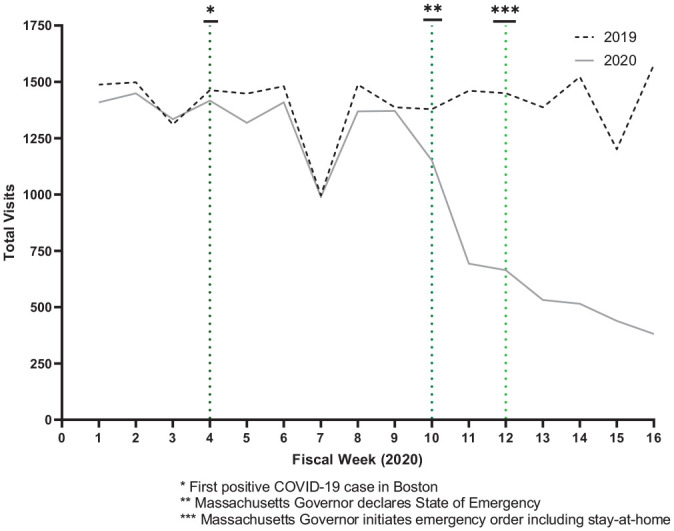
Total visits across 10 otolaryngology practices during the first 16 fiscal weeks of 2020.

Aggregate week-by-week visit and procedure data for 20 general otolaryngology physicians across 10 practices in Massachusetts are shown in Table 2. Total visits in weeks 1 to 10 of 2019 and weeks 1 to 10 of 2020 were not statistically different, while a significant decrease of 62.5% of visits is observed when comparing weeks 11 to 16 of 2019 and weeks 11 to 16 of 2020 (*P* < .0001; 1432.8 [95% CI, 1295.7-1570.0] vs 537.3 [95% CI, 408.9-655.8]). These numbers were reflected in a decrease in both consult visits and established visits. Week 10 in 2020 corresponds to the time that the Massachusetts governor declared a statewide State of Emergency (on March 10, 2020), while week 12 corresponds to the statewide emergency stay-at-home recommendation (on March 23, 2020). These events closely corresponded to a decrease in total visits (**Figure 1**). Notably, the first coronavirus case was diagnosed in Boston on February 1, 2020, 6 weeks prior to an observed decline in clinical volume.

**Table 2.** Visits Across 10 Otolaryngology Private Practices for Weeks 1 to 16 of 2019 and 2020.^a^

Characteristic	Weeks 1-10 in 2019	Weeks 11-16 in 2019	Weeks 1-10 in 2020	Weeks 11-16 in 2020	*P* value 2019, 1-10 vs 11-16	*P* value 2020, 1-10 vs 11-16	*P* value, 1-10 in 2019 vs 2020	*P* value, 11-16 in 2019 vs 2020
Total visits	1393.9 (1284.8- 1503.0)	1432.8 (1295.7- 1570.0)	1321.6 (1219.0- 1424.2)	537.3 (408.9- 665.8)	.61	**<.0001**	.29	**<.0001**
In-person visits	1393.9 (1284.8- 1503.0)	1432.8 (1295.7- 1570.0)	1320.8 (1218.0- 1423.6)	243.5 (56.5-430.5)	.61	**<.0001**	.28	**<.0001**
Virtual visits	0	0	0.8 (.06-1.5)	293.8 (167.4-420.3)	NA	**<.0001**	.025	**<.0001**
Consult visits	475.5 (430.8-520.2)	464.1 ± 87.4	453.8 (372.4-556.0)	159 (107.3-210.7)	.77	**<.0001**	.44	**<.0001**
Established visits	625.6 (561.3-690.0)	627 ± (527.8-726.2)	659.7 (594.3-725.0)	279.1 (234.2-324.1)	.98	**<.0001**	.41	**<.0001**

Abbreviation: NA, not available.

a Values represent average weekly volume within time period (95% CIs in parentheses). *P* value < .05 (bolded) indicates statistical significance (95% confidence).

**Figure 2.**
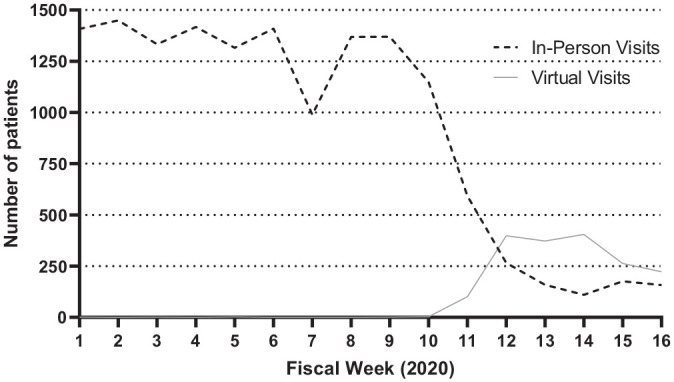
In-person visits and virtual visits across 10 otolaryngology practices during the first 16 fiscal weeks of 2020.

**Figure 2** highlights that while total visits declined precipitously during weeks 11 to 16 of 2020, they were partially but incompletely replaced by virtual visits. After no virtual visits were performed in 2019, and fewer than 1 virtual visit was performed on average weekly in the first 10 weeks of 2020 (0.8; 95% CI, 0.06-1.5), weeks 11 to 16 of 2020 had an average of 293.8 (95% CI, 167.4-420.3) virtual visits per week. The difference in both cases relative to weeks 11 to 16 of 2020 was statistically significant (*P* < .0001 for both). By week 12, virtual visits comprised a greater proportion of overall visits than in-person visits, which continued through week 16.

**Figure 3.**
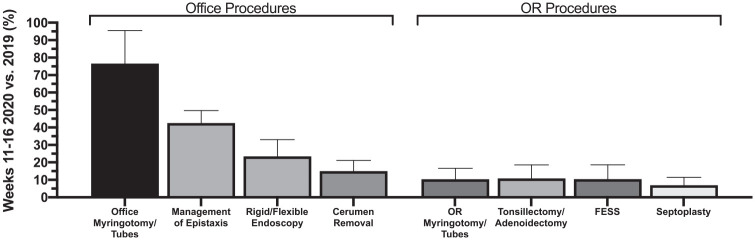
Relative (percentage) of office procedures and OR procedures across 10 otolaryngology practices in fiscal weeks 11-16 of 2020 compared to fiscal weeks 11-16 of 2019.

Of additional interest is volume of clinic and operating room (OR) procedures performed by general otolaryngologists, listed in **Table 3**. Significant decreases were observed for all evaluated OR procedures in weeks 11 to 16 of 2020 compared to 2019 data, including functional endoscopic sinus surgery (90% decrease), myringotomy with or without tube placement (90% decrease), tonsillectomy and/or adenoidectomy (89% decrease), and septoplasty (93% decrease) (*P* < .001). Clinic procedures including cerumen removal (85% decrease), control of epistaxis (58% decrease), and flexible and rigid endoscopy (77% decrease) saw corresponding significant decreases in 2020 (*P* < .001), while clinic myringotomy did not decrease to a significant degree (**Figure 3**).

**Table 3.** In-Office and OR Procedures in 10 Otolaryngology Private Practices for Weeks 1 to 16 of 2019 and 2020.^a^

Characteristic	Weeks 1-10 in 2019	Weeks 11-16 in 2019	Weeks 1-10 in 2020	Weeks 11-16 in 2020	*P* value 2019, 1-10 vs 11-16	*P* value 2020, 1-10 vs 11-16	*P* value, 1-10 in 2019 vs 2020	*P* value, 11-16 in 2019 vs 2020
In-office procedures								
Endoscopic exam	271.6 (235.8- 307.4)	258.8 (200.0-317.7)	298.1 (261.8 334.4)	55.7 (6.0-105.3)	.64	**<.0001**	.26	**<.0001**
Epistaxis management	28.6 (21.9-35.3)	25 (16.8-33.2)	27.7 (23.3-32.1)	10.5 (4.6-16.4)	.44	**.0001**	.80	**.004**
Cerumen removal	156.3 (134.6-178)	161.8 (119.6-203.0)	171.4 (148.3-194.5)	23.1 (0-46.7)	.76	**<.0001**	.29	**<.0001**
Office myringotomy with or without tubes	7.9 (4.9-10.9)	4.2 (2.0-6.3)	9.7 (6.2- 13.2)	2.5 (1.1-3.9)	.07	**.004**	.39	.13
OR procedures								
Septoplasty	13.6 (10.9-16.3)	13 (7.9-18.1)	12.8 (15.5-1.9)	0.7 (0-1.9)	.79	**<.0001**	.64	**.0001**
OR myringotomy with or without tubes	24.5 (17.0-32.0)	23.8 (15.1-32.5)	25.3 (19.7-30.9)	2 (0.5.0)	.89	**<.0001**	.85	**.0001**
Tonsillectomy and/or adenoidectomy	18.2 (12.0-24.4)	15.2 (9.5-20.9)	14.6 (11.3-17.9)	1.2 (0-3.3)	.46	**<.0001**	.26	**.0001**
FESS	6.7 (4.7-8.7)	7 (4.5-9.5)	9.1 (6.7-11.5)	0.7 (0-1.9)	.83	**<.0001**	.10	**.0002**

Abbreviations: FESS, functional endoscopic sinus surgery; OR, operating room.

a Values represent average weekly volume within time period (95% CIs in parentheses). *P* value < .05 (bolded) indicates statistical significance (95% confidence).

## Discussion

COVID-19 caused tremendous disruption in the provision of health care services in Massachusetts. Following international and federal recommendations, significant public policy changes were made to adapt to the new challenge. Shortly after the first positive COVID-19 case in Boston, the Massachusetts governor declared a state of emergency on March 10, 2020 (week 10), a day before the WHO declared COVID-19 a pandemic.^8,9^ Three days later, the American College of Surgeons urged physicians to postpone or cancel all nonurgent clinical visits and elective surgeries as well.^10^ Then, on March 23, 2020 (week 12), the Massachusetts governor announced an emergency order, including closure of all “nonessential” physical workplaces and a stay-at-home order for at least 2 weeks.^8^ These recommendations and government orders substantially altered many practices’ operations.

While this pandemic affects all medical fields, otolaryngologists are among the groups thought to have the highest risk of contracting COVID-19^11^ due to proximity of clinical examination to the upper airway.^4^ Beyond anatomical exposure, common otolaryngology procedures also can generate airborne particulate through powered instrumentation, potentially increasing exposure risk if COVID-19 can be transmitted in this fashion.^12-14^ COVID-19 also is associated with high rates of respiratory failure in patients, often requiring urgent airway management by otolaryngologists, in which procedures like intubation and tracheostomy are particularly high risk.^15,16^ Finally, the disease has been shown to transmit via asymptomatic carriers, facilitating interpersonal spread in a busy clinical practice.^17^

New guidelines, combined with public apprehension of in-person health care, resulted in significant changes in practice patterns, finances, and patient outcomes for private practices. Due to COVID-19, 9 of 10 clinics represented in this data set cut back working hours, with an average 75.5% decrease. The hour reduction placed substantial constraints on prompt patient care. It also led to financial constraints for practices for maintaining fixed and overhead expenses. As a result, 8 practices furloughed personnel, and 2 practices had permanent layoffs. In response to the decrease in revenue, 7 clinics applied for PPP assistance. Despite 2 applications being delayed, all PPP recipients reported benefits from the funding for obviating additional layoffs. These outcomes highlight the necessity and efficacy of federal assistance to sustain otolaryngology practices during this challenging time.

Availability of otolaryngology services differed starkly before and after COVID-19. Following the Massachusetts declaration of a state of emergency, practices providing in-person audiology decreased by 80%, with none providing remote or nonemergent appointments. During this hiatus of audiology services, communities were at risk for missed sudden sensorineural hearing loss diagnoses and subsequent expedient treatment. In addition, patients with nonemergent problems were left without audiologic care at a time during a period of widespread mask use, which could further strain patients’ ability to communicate. Only 6 of 10 practices continued performing rigid or flexible endoscopy. The inherent perceived danger of endoscopy prompted all practices included to implement stricter selective criteria. Reduction in the availability of endoscopy, combined with broader exclusion criteria, may have delayed or decreased the accuracy of diagnosis of a number of time-sensitive conditions, including head and neck malignancy, and this would be a worthwhile focus of further investigation.

Our study found a precipitous decline in the utilization of private practice total visits, established visits, and new patient visits within otolaryngology, most notably in weeks 11 to 16 of 2020, relative to both early 2020 (weeks 1-10) and weeks 11-16 of 2019. The greater decline in total visits of weeks 11 to 16 in contrast to weeks 1 to 10 suggests the decrease is most aligned with the influence of governmental policies, as opposed to direct natural pandemic progression. Comparisons can be drawn to the severe acute respiratory syndrome outbreak in 2003, where the Prince of Wales Hospital in Hong Kong observed a 59% decrease in outpatient clinic attendance, a 79% decrease in number of operations performed, and an 84% decrease in daily hospital admission rates.^18^ These decreased patient visits were attributed to cancellation of nonessential elective surgeries, public recommendations to stay-at-home, and patients canceling appointments, similar to what was more recently observed in Massachusetts.^18^

Based on our data, OR procedures had a higher percentage decline than office procedures, which is likely due to several factors. Canceling OR procedures preserves PPE, which was critically low in supply nationwide in early phases of the COVID-19 response. Furthermore, OR procedures necessitate higher potential inpatient admissions, which can further exhaust limited medical personnel and resources. Another factor in the decline in OR cases is that the hospital regulates availability of OR time, based on government and health care system recommendations. Findings may suggest response to COVID-19 had a greater impact on canceling OR procedures than clinic procedures, seen most prominently in our myringotomy/tympanostomy tube placement data. Office myringotomy remained stable from pre–COVID-19 levels during weeks 11 to 16 of 2020, while OR myringotomy rates dropped by almost 90%. This finding is likely most applicable to interventions without upper airway involvement to mitigate aerosol-generating procedures. The rise of telemedicine also provided another significant tool to prescreen patients and identify individuals who may be candidates for office-based procedures.^19^

Although the United States has established telemedicine programs offered by more than 50 health systems,^19^ pre–COVID-19 telemedicine users were limited to medical specialties with low barriers to entry and easily digitized encounters, such as psychiatry and dermatology.^20,21^ Prior to the COVID-19 pandemic, otolaryngology as a field rarely used telemedicine, with significant uptake noted alongside policy changes that were announced in March 2020 by both Medicare and private payors to implement less restrictive and more remunerative coverage policies for telemedicine.^22,23^ Our findings in this study corroborate this trend seen in the field of otolaryngology as a whole. Before 2020, virtual visits were not a part of any of the otolaryngology practices that we studied, and yet, the number of telemedicine visits surpassed in-person visits consistently after week 12 of 2020. This is not unique to the United States; Hong Kong hospitals used technology, specifically Zoom, a virtual meeting program, to continue regular checkup visits while triaging possible patients in need for operations.^24^ Not only does telemedicine allow physicians to assess patients from a safe distance, but it also likely decreased the buildup of canceled or postponed patient flow post–COVID-19.

Our study has several limitations, first and foremost its retrospective nature from a single source of data for multiple otolaryngology groups. Our inclusion group was from Massachusetts only, which restricts this study’s implications for other geographic regions. In addition, 20 general otolaryngologists were represented in our data set from 10 practices, implying that one practice’s decision may potentially affect outcomes for multiple physicians. Only the first 4 months of 2020 were selected for examination based on available data at the time of manuscript preparation. Lastly, this study specifically focuses on the field of otolaryngology and its unique relationship with COVID-19 during this unprecedented time; other specialties may have seen varying alterations in the provision of clinical and surgical care. A key strength of this study is that it focuses on the COVID-19 experience of general otolaryngologists rather than their academic counterparts, the former of which have been excluded from reporting to date.

## Conclusion

COVID-19 had a multifaceted impact on otolaryngology practices in Massachusetts. To our knowledge, this is the first article to quantify this impact within otolaryngology private practices. Significant declines in provision of otolaryngology services aligned with Massachusetts government’s public health policy changes in response to COVID-19. The combination of limited personnel and PPE, as well as suspension of nonessential office visits and surgeries, led to a decrease in total office visits and even higher decrease in OR procedures. This reduction in otolaryngology services was only partially replaced by telemedicine visits, and therefore, overall delivery of otolaryngology care was substantially diminished due to COVID-19.
